# Gene Therapy for Chronic HBV—Can We Eliminate cccDNA?

**DOI:** 10.3390/genes9040207

**Published:** 2018-04-12

**Authors:** Kristie Bloom, Mohube Betty Maepa, Abdullah Ely, Patrick Arbuthnot

**Affiliations:** Wits/SAMRC Antiviral Gene Therapy Research Unit, School of Pathology, Faculty of Health Sciences, University of the Witwatersrand, Private Bag 3, Johannesburg, WITS 2050, South Africa; betty.maepa@wits.ac.za (M.B.M.); abdullah.ely@wits.ac.za (A.E.); patrick.arbuthnot@wits.ac.za (P.A.)

**Keywords:** hepatitis B virus, covalently closed circular DNA, gene therapy, epigenetic modification, designer nucleases

## Abstract

Chronic infection with the hepatitis B virus (HBV) is a global health concern and accounts for approximately 1 million deaths annually. Amongst other limitations of current anti-HBV treatment, failure to eliminate the viral covalently closed circular DNA (cccDNA) and emergence of resistance remain the most worrisome. Viral rebound from latent episomal cccDNA reservoirs occurs following cessation of therapy, patient non-compliance, or the development of escape mutants. Simultaneous viral co-infections, such as by HIV-1, further complicate therapeutic interventions. These challenges have prompted development of novel targeted hepatitis B therapies. Given the ease with which highly specific and potent nucleic acid therapeutics can be rationally designed, gene therapy has generated interest for antiviral application. Gene therapy strategies developed for HBV include gene silencing by harnessing RNA interference, transcriptional inhibition through epigenetic modification of target DNA, genome editing by designer nucleases, and immune modulation with cytokines. DNA-binding domains and effectors based on the zinc finger (ZF), transcription activator-like effector (TALE), and clustered regularly interspaced short palindromic repeat (CRISPR) systems are remarkably well suited to targeting episomal cccDNA. This review discusses recent developments and challenges facing the field of anti-HBV gene therapy, its potential curative significance and the progress towards clinical application.

## 1. Introduction

Viral hepatitis accounts for up to 1.34 million deaths per year and remains the major cause of morbidity and mortality from cirrhosis and hepatocellular carcinoma [1]. Hepatitis B virus (HBV) infections contribute significantly to the global health problem and, with an estimated 257 million people chronically infected, it is a major health priority. More than 50 years have passed since the discovery of the Australia antigen [2], and while valuable progress has been made in vaccine and antiviral development, there is still no reliable cure for HBV infection. Worldwide prophylactic vaccination programs have reduced the prevalence of HBV in children under the age of five, but inadequate coverage in hyper-endemic African countries means that prevalence remains high in some countries [1]. Management of chronic HBV infection involves use of immune modulators or direct-acting antivirals, in the form of interferons or nucleoside/nucleotide analogs (NAs). The rationale for combining these therapies is to manage both immune dysregulation as well as viral pathogenesis. Currently, approved therapeutics include interferon α, pegylated interferon α, lamivudine, telbivudine, adenovir dipivoxil, entecavir, tenofovir disoproxil fumarate, and tenofovir alafenamide. Recommendations for first-line monotherapies are pegylated interferon α and the NAs with high barriers to resistance, which are entecavir, tenofovir disoproxil fumarate, and tenofovir alafenamide [3,4,5]. Guidelines for administration of combination therapies sometimes vary as a result of conflicting opinions about long-term efficacy, influence of patient selection, and whether simultaneous or sequential administration is favored (reviewed by [6,7]).

The critical limitation of licensed therapeutics is their inability to reliably achieve a virological cure [3]. While NAs inhibit posttranscriptional stages of viral replication, they do not target the stable episomal covalently closed circular DNA (cccDNA). This key HBV replication intermediate forms a minichromosome in the nucleus of hepatocytes [8,9,10], and may undergo epigenetic modifications [11,12,13]. The hepatitis B X protein (HBx) plays a role in stabilizing cccDNA by inactivating components of the structural maintenance of chromosomes (SMC) complex [14]. The cccDNA associates with host transcription factors and viral proteins to enable viral gene expression and replication. Associations with factors regulating methylation or heterochromatin formation may also render cccDNA inactive, and lead to persistent latent or occult HBV infections. Cessation or interruption of antiviral therapy, development of viral escape mutants, or immunodeficiency could all lead to reactivation of HBV replication, highlighting the need for cccDNA-specific therapies. This review focuses on recent advances aimed at generating HBV-targeting designer nucleases, epigenetic modifications to the viral DNA and nucleic acid-based immune modulation to treat chronic HBV infection.

## 2. Hepatitis B Virus Therapies Under Development

Several novel anti-HBV therapeutics are in preclinical development or early clinical trial (reviewed by [15,16]). Most candidate drugs are small molecule drugs designed to impede various stages of HBV replication. Affordable next-generation NAs, which inhibit the viral polymerase with reduced toxicity and higher barriers to HBV resistance are currently the preferred first-line of therapy. Other direct-acting antivirals include HBV core protein allosteric modulators [17,18], HBV surface antigen (HBsAg) release inhibitors [19,20] and nucleic acid polymers which also inhibit viral entry [21]. With the discovery that the sodium taurocholate co-transporting polypeptide (NTCP) facilitates HBV entry into hepatocytes [22], peptide inhibitors such as Myrcludex-B (NCT02881008 and NCT02888106) are also being developed for therapeutic application [23]. Another popular host-related strategy has been to recondition the immune system using interferons, cytokines, and peptides as immune modulators which are discussed in more detail below (Section 3.3).

Use of gene therapy to disable HBV replication has shown promise and generated considerable interest. Different strategies that have been employed include HBV-specific gene silencing, gene editing, epigenome modification, and nucleic acid-based vaccination. Harnessing the RNA interference (RNAi) pathway is a well-established strategy that has been used extensively to silence genes of HBV. RNAi is an endogenous gene regulatory pathway that can be reprogrammed by exogenous RNA molecules. Feasibility of using RNAi to treat HBV has been established and extensively reviewed elsewhere [24,25] and will not be covered in detail here. Expressed or synthetic antiviral sequences may mimic primary microRNAs, precursor microRNAs, or mature microRNAs (miRs) [26,27,28,29]. Because of easier large-scale production, dose control and delivery to the cytoplasmic site of action, development of synthetic short interfering RNAs (siRNAs) has advanced rapidly. These simulate mature miRs, and are now in Phase 2 clinical trials. Anti-HBV siRNA formulations with impressive antiviral activity include ARC-520 (NCT02065336), ALN-HBV (NCT02826018), and ARB-001467 (NCT02631096). However, since their effect is transient, repeated administration of siRNAs will be required for long term anti-HBV efficacy. To increase durability of an anti-HBV effect expressed HBV-targeting sequences have been developed. For example, adeno-associated viral vectors (AAVs) have been used to deliver cassettes that express HBV-targeting primary microRNA mimics [30]. Safe and sustained inhibition of HBV replication in HBV transgenic mice indicates that this approach has potential for clinical translation. Strategies targeting host factors have also shown significant promise in reducing cccDNA levels. RNAi-mediated gene silencing of tyrosyl-DNA-phosphodiesterase 2, a DNA repair enzyme thought to mediate polymerase release from the relaxed circular DNA, delays its conversion to cccDNA in HepG2 cells [31]. A recent study has also demonstrated that silencing the expression of pre-mRNA processing factor 31, an HBx-interacting partner, reduces cccDNA levels in HepAD38 cells [32].

## 3. Gene-Based Therapies to Target Covalently Closed Circular DNA

While the HBV therapeutic landscape is vast, few approaches are being developed to disable cccDNA directly. Targeted mutagenesis by sequence-specific RNA-guided nucleases (RGNs) and proteins has thus generated considerable interest, as the technology potentially provides the means to cure HBV infection by permanently disabling cccDNA [33,34].

### 3.1. Designer Nucleases 

**Figure 1:**
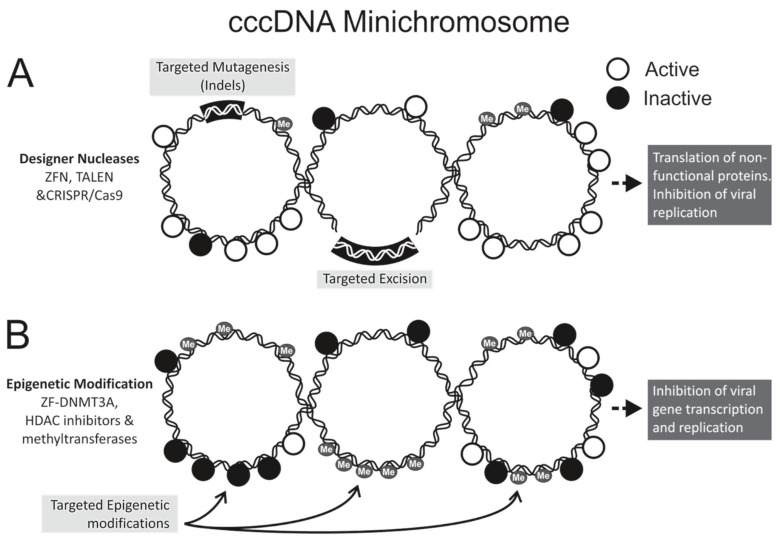
Strategies for hepatitis B virus (HBV) gene editing and epigenome modifications. The covalently closed circular DNA (cccDNA), which may be methylated, forms a minichromosome with transcriptionally active (open circles) and inactive (closed circles) chromatin. (**A**) Designer nucleases cleave at pre-defined sequences within the HBV genome to effect targeted mutagenesis. Employing multiple nucleases to digest different sequences may lead to targeted excision. Mutated cccDNA may be transcribed, but mutant viral proteins cannot carry out viral replication; (**B**) Epigenetic modification involves conversion of actively transcribed DNA to a transcriptionally inactive state without altering the nucleotide sequence the viral DNA. Targeted modifications occur when DNA-binding domains guide epigenetic effectors to pre-defined sequences of cccDNA. Histone modification and cccDNA methylation may affect epigenetic modifications by acting directly on the cccDNA or on associated histone proteins. Indels: insertions and deletions; ZFN: zinc finger nuclease; TALEN: transcription activator-like effector nuclease; CRISPR/Cas: clustered regularly interspaced palindromic repeats with CRISPR-associated protein; HDAC: histone deacetylase; DNMT: DNA methyltransferase; Me: methyl.

Designer nucleases have dominated the anti-HBV gene editing field, with zinc finger nucleases (ZFNs), transcription activator-like effector nucleases (TALENs), and clustered regularly interspaced short palindromic repeats (CRISPR) with CRISPR-associated (Cas) systems all showing antiviral efficacy (Table 1). Nucleases act by inducing double stranded breaks at a pre-defined target site within the HBV genome (Figure 1A). By exploiting the host cells’ error-prone non-homologous end joining repair machinery, targeted mutagenesis is realized. HBV cccDNA is a primary candidate for nuclease gene editing, owing to its episomal minichromosome configuration and limited sequence plasticity. The compact viral genome and overlapping reading frames restrict development of escape mutants, despite the low fidelity of the viral reverse transcriptase [35]. Insertions and deletions (Indels) within the viral genome may give rise to aberrant or truncated proteins, which in turn interfere with viral replication.

Zinc fingers (ZFs) are abundant multifunctional mammalian proteins that occur naturally as transcription factors. By exploiting the specific targeting of nucleotide triplets of single ZFs, these proteins may be engineered to form arrays with defined DNA binding properties [36]. Addition of a *Fok*I effector domain at the C-terminus of a ZF yields an engineered nuclease that cuts one strand of a DNA duplex. ZFN dimers, with cognates on complementary strands of the DNA duplex, may thus be used to create double stranded breaks [37]. Anti-HBV ZFNs were first described in a proof-of-concept experiment where 36% of plasmid-derived viral sequences were disrupted in a cell culture model of viral replication [38]. More recently Weber et al. reported on delivery of self-complementary adeno-associated viral vectors (scAAVs) encoding ZFNs targeting the polymerase/*X* (1), polymerase/core (2) and polymerase (3) viral open reading frames (ORFs) [39]. Using inducible liver-derived HepAD38 cells to mimic natural HBV infection [40], ZFN pairs 1, 2 and 3 cleaved 9.8, 34, and 28% of the viral targets respectively. These results were confirmed using single molecule real time sequencing which additionally identified potential off targets, albeit at low frequencies. Interestingly ZFN pair 2 resulted in the highest detectable targeted disruption, yet it was also found to be cytotoxic. Only ZFN pair 3 showed antiviral efficacy but cleavage of cccDNA could not be verified.

As with ZFNs, TALENs are dimeric engineered nucleases that comprise a DNA-binding protein fused to an endonuclease domain. The transcriptional activator-like effector (TALE) is derived from the *Xanthomonas* bacteria where individual repeat domains comprising 33–35 amino acids recognize a single base pair [41,42]. The nucleotide binding specificity of these repeats is predetermined by repeat-variable di-residues (RVDs) located at amino acid positions 12 and 13 [43,44]. Linking multiple repeats in a defined order generates engineered TALEs with highly specific DNA binding properties. Unlike with ZFNs, nucleotide binding affinity of each monomer comprising the DNA binding domain is not influenced by a neighboring unit. Our group first described antiviral efficacy of engineered TALENs on HBV cccDNA in cultured cells and inhibition of viral replication in a murine model [45]. TALEN dimers designed to bind within the *surface* (S) and *core* (C) ORFs showed optimal cleavage activity in the HepG2.2.15 cell line without measurable cytotoxicity. Importantly, cccDNA targeted disruption frequencies of 35% and 12% were achieved with the S and C TALEN pairs respectively. Using the murine hydrodynamic injection (HDI) model, co-administration of HBV replication-competent plasmids with TALEN-encoding sequences demonstrated in vivo antiviral efficacy of the nucleases and there was no evidence of liver toxicity. A significant and substantial reduction in serum concentrations of HBsAg and circulating viral particle equivalents was observed in TALEN-treated mice, and targeted mutagenesis of up to 87% was achieved. Deep sequencing verified large deletions in viral DNA, which were likely to inactivate HBV replication. A subsequent study by Chen et al. confirmed the cccDNA-specific antiviral potential of TALENs designed to target conserved regions within the polymerase (RNaseH sequence) and C ORFs [46]. This was achieved in liver-derived Huh7 cells transfected with linear viral sequences that generate cccDNA and recapitulate HBV replication [11,12]. A reduction in viral protein expression was observed across genotypes A, B, C, and D, emphasizing the applicability of anti-HBV TALENs to a variety of viral isolates. Moreover, a synergistic effect was shown when combining IFN-α with core TALENs, to result in an approximately 60% reduction in copies of cccDNA. In another study, co-transfection of linear donor sequences encoding trimeric gene silencers significantly augmented anti-HBV efficacy of S or C TALEN pairs in HepG2.2.15 cells [47]. This approach exploited the homology directed repair pathway to introduce the artificial primary microRNA-encoding sequences directly into viral DNAs. In doing so, the viral genome may be permanently disrupted and after homologous recombination, HBV DNA transcribes an antiviral sequence from its own rearranged genome.

Use of RGNs is now the most popular method of inactivating HBV gene expression. This bacterial CRISPR/Cas9 system relies on an RNA guided DNA binding domain with associated Cas9 endonuclease [48]. These RGNs typically comprise a CRISPR RNA (crRNA) with sequences that are complementary to a pre-defined DNA target sequence and a *trans*-activating crRNA (tracrRNA). Annealing of the RNA to its cognate enables Cas9-mediated double-stranded target DNA cleavage. Fusing crRNA and tracrRNA to form a single guide RNA (gRNA) has further simplified the system, which has made RGNs the easiest nucleases to engineer. Since first reported by Lin et al. in 2014 [46], more than 16 publications have described anti-HBV efficacy of RGNs [49,50,51,52,53,54,55,56,57,58,59,60,61,62,63,64,65] (Table 1). Targeted mutagenesis of cccDNA in mammalian cell cultures was first demonstrated by Seeger and Sohn [49], who designed CRISPR/Cas9 constructs to bind the conserved enhancer II/core promoter and precore ORFs. Subsequent studies confirmed that single or multiple gRNAs spanning the entire HBV genome cleaved cccDNA to cause indel formation and resultant reduced viral protein expression [50,51,52,54,55,56,59,62,63,64,65]. However, methods used to detect mutagenic events vary greatly between individual studies, which makes it difficult to compare efficiency of different gRNA constructs directly. Nevertheless, as with all designer nucleases, targeting conserved regions is advantageous as antiviral efficacy is achieved across different genotypes [53,55]. A second study by Seeger and Sohn used next generation sequencing to map CRISPR/Cas9-induced cccDNA mutations [58]. The majority of indels were identified as small deletions when extremely high targeted cleavage (up to 90%) was achieved. Although off-target cleavage may be frequently associated with CRISPR/Cas9 action [66,67,68], data on the potential of HBV RGNs to create nonspecific mutations is scarce. Using multiple gRNAs to improve antiviral efficacy [46] or to excise integrated viral DNA fragments (Figure 1A) [62] may exacerbate this issue. As expected off-target cleavage events were detected by deep sequencing when multiple gRNAs were used [57,62]. However, other studies report no detectable genotoxic activity [54,61,63], which may be a result of differences between gRNA design and properties of the viral target sequences. Replacing the Cas9 endonuclease with engineered nickases may improve specificity of HBV RGNs. With this configuration, gRNA and Cas9 nickases comprising heterodimers are required to effect double stranded DNA cleavage [51,57]. Another challenge with the CRISPR/Cas9 system relates to delivery of the combined gRNA and the large Cas9 endonuclease. Sequences encoding these two components exceed the transgene capacity of the popular AAVs. A recent publication by Scott et al. demonstrated that the smaller Cas9 derived from *Staphylococcus aureus* (Sa), together with expression cassettes encoding short gRNAs targeting the HBV surface ORF, could be packaged into single stranded AAVs (ssAAV) [63]. Efficient delivery and expression of the RGN-encoding ssAAVs in HepG2.2.15 and hNTCP-HepG2 cells resulted in decreased viral replication, target specific cccDNA cleavage, and reduced cccDNA copy numbers. Analysis of sequences at predicted off target sites did not reveal non-specific mutagenesis.

### 3.2. Epigenetic Gene Silencing

Natural epigenetic modification of DNA may silence gene expression and is a host defence mechanism against expression of viral genes. Epigenetic modification is a stable and heritable gene regulatory mechanism found in many different organisms. It involves chemical alteration of DNA or associated proteins without changing the encoded genetic information. It is involved in typical cell development and multiple normal or abnormal cellular responses (reviewed by [69]). There is accumulating evidence to indicate that epigenetic machinery controls transcription of HBV cccDNA (Figure 1B), and contributes to the outcome of chronic HBV infection [70,71,72]. Use of exogenous epigenetic modifiers has thus attracted interest as a mode of disabling cccDNA. Epigenetic modifications include histone acetylation or deacetylation, histone methylation or demethylation, cccDNA methylation, and cccDNA minichromosome acetylation (reviewed by [73,74]). In cooperation with viral factors such as HBx and the core antigen (HBc), the major epigenetic modifiers of HBV DNA include histone acetyltransferases/deacetylases (HATs/HDACs) [12], lysine methyltransferases [75], protein arginine methyltransferases [70,76], and DNA methyltransferases (DNMTs) [77].

Hypoacetylation of cccDNA-bound histone 3/4 results in low HBV viremia in hepatitis B patients, whereas hyperacetylation increases HBV replication [11]. HDAC inhibitors have been shown to suppress cccDNA transcription in duck hepatitis B virus (DHBV) [82,83]. Methylation of arginine 3 on cccDNA-bound histone 4 prevents RNA polymerase binding and transcription in an arginine methyltransferase 5 and HBc-dependent manner [70,76]. Transcriptional inhibition by methylation can be direct, by blocking binding of transcriptional factors or RNA-polymerase loading, as well as indirect, by recruiting histone-modifying and chromatin remodelling complexes to the methylated DNA (reviewed by [69]). Epigenetic gene silencing may facilitate reduction of viral reservoirs through normal hepatocyte turnover and prevention of replenishment of the cccDNA pool. Furthermore, epigenetic modifications may accelerate cccDNA decay, as was shown following IFN-α treatment of DHBV infections [82]. HBV cccDNA contains three CpG islands: island I, island II and island III [84,85]. Island I overlaps the start site of the *S* gene, island II encompasses enhancer I, the *HBx* gene promoter and the core promoter, whereas island III harbors the Sp1 promoter and start codon of the polymerase gene. Methylation of island I is rare and variable across genotypes, whereas methylation of island II and III is more conserved. Island III methylation is associated with reduced serum HBsAg concentrations in chronically infected patients and correlates with hepatocarcinogenesis. Island II methylation is associated with reduced pregenomic RNA (pgRNA) transcription, viral replication and viremia [72,86]. In vitro studies showed that several DNMTs are upregulated in response to HBV infection, leading to viral DNA methylation, decreased HBV gene expression, and diminished viral replication [77,87]. Importantly, similar reduction in viral gene expression and viremia was observed in human tissue samples with methylated HBV DNA [85,88,89].

Despite the importance of HBV DNA epigenetic modification for disease progression, evidence supporting the feasibility of using epigenetic modifiers against HBV is currently limited. Few studies have taken advantage of the sequence-specific binding domains of designer nucleases for their repurposing as epigenetic silencers. By replacing a nuclease domain of a designer nuclease with an epigenetic modulator, ZFs, TALEs, and CRISPR/Cas have usually been modified for epigenetic editing of endogenous genes (reviewed by [90]). However, this strategy is theoretically applicable to targeted epigenetic modification of HBV DNA. One study reported successful epigenetic editing of HBV DNA after fusing the catalytic domain of DNMT3a to a ZF that targeted the *HBx* promoter sequence [79]. The engineered sequence caused methylation of targeted CpG sites, downregulation of viral mRNAs and proteins, and a decrease in viral replication in cell culture and in HBV transgenic mice. Repressors based on the Krüppel associated box (KRAB) domain have also been investigated as ZF [80] and TALE [81] fusions. Although these KRAB-repressors inhibit viral replication, verification of heterochromatin formation, which is important to achieve permanent gene silencing, remains to be confirmed. Studies demonstrating antiviral efficacy and sustainability of epigenetic editors on HBV replication are preliminary. However, investigations aimed at developing the approach to treat other chronic viral infections, such as HIV [91], are more advanced. Moreover, Food and Drug Administration (FDA) approval of HDAC inhibitors for cancer treatment [92] supports the notion that epigenetic editing has clinical potential for treatment of HBV infection.

### 3.3. Immune Modulation for Covalently Closed Circular DNA Attenuation

Administration of recombinant IFN-α or its pegylated derivatives remains the only immunomodulatory drugs licensed for management of chronic HBV infection. IFN-α is therefore the only licensed anti-HBV therapy capable of eliminating cccDNA. Immunomodulation has been shown to augment innate and adaptive immune responses against the virus. Stimulating T-cell-mediated elimination of infected hepatocytes, and indirectly cccDNA, is thus a promising immune-based strategy to achieve functional cure from HBV infection. However, the success rate of IFN therapy remains low and side effects represent a major shortcoming of this therapy. Gene therapy to enable durable expression of immune modulators may be useful to attenuate cccDNA.

Expression of *IFN-α*-encoding sequences in the liver has been explored as a means of improving anti-HBV efficacy and reducing side effects of conventional IFN-α treatment. One of the first studies explored expression of murine IFN-α2 under transcriptional control of the liver-specific transthyretin promoter [93]. The IFN-α expression construct was delivered to the livers of mice using a helper-dependent adenovirus (HDAd), and the interferon response genes 2′,5′-oligoadenylate synthetase and tumor necrosis factor α (TNF-α) were effectively induced. As a surrogate for assessing anti-HBV potential of this system the authors challenged mice with a murine coronavirus, MHV-2. Mice pre-treated with the HDAd carrying IFN-α were protected from infection and did not exhibit any toxic side effects. Fiedler et al. assessed usefulness of a gene therapy-based approach to express IFN-α or IFN-γ in a woodchuck hepatitis virus (WHV) model of HBV infection [94]. Sequences encoding woodchuck IFN-α or IFN-γ (wIFN-α or wIFN-γ) were delivered to woodchucks with an HDAd. In animals chronically infected with WHV, viral replicative intermediates in the liver and serum were considerably diminished. This was a significant finding as WHV maintains very high viral loads in its host, much higher than in chronic HBV carriers. Furthermore, no obvious side effects were observed and wIFN-α expression lasted for at least a year. In contrast to the promising results achieved with wIFN-α, expression of wIFN-γ did not have a significant effect on WHV replication. This differs from the results of Dumortier et al. who demonstrated that IFN-γ expression was able to limit HBV replication in mice [95].

Subsequent work assessed utility of AAV vector for delivery of interferon expression cassettes. Berraondo et al. tested intrahepatic and intramuscular expression of AAV8-delivered wIFN-α5 following administration to woodchucks chronically infected with WHV [96]. Interferon was readily expressed at both sites of delivery, but expression in the liver was superior. Furthermore, only expression of interferon from the liver was associated with an antiviral response. Although the higher vector dose had a more pronounced antiviral effect, it was also associated with greater toxicity. The woodchuck with the greatest decrease in viremia exhibited such severe side effects that it required euthanasia prior to completion of the study. Another limitation of the approach was the transient nature of the antiviral effect, which was attributed to immune-mediated clearance of AAV-infected hepatocytes. To improve their delivery system Berraondo et al. developed a modified IFN-α which was fused to sequences encoding apolipoprotein A (InterApo) [97]. Initial assessment demonstrated that InterApo exhibited an improved safety profile while maintaining antiviral efficacy in HBV transgenic mice. In the woodchucks chronically infected with WHV, AAV-delivered InterApo was well-tolerated but showed little efficacy. This was attributed to the high WHV load in woodchucks with chronic hepatitis. Pre-treatment of woodchucks with the NA entecavir with subsequent administration of AAV-InterApo facilitated immune-mediated clearance. However, the antiviral effect was transient and viral rebound to pre-treatment levels eventually occurred.

Further evaluation of gene therapy-based immune modulation explored antiviral effects of sequences encoding interleukins. Crettaz et al. assessed efficacy of HDAd-delivered interleukin-12 (IL-12) in the WHV model [98]. The authors used a murine *IL-12* sequence, under transcriptional control of an inducible promoter, and delivered the cassette directly to the liver with the viral vector. Interestingly, woodchucks with a viral load lower than 10^10^ viral genome equivalents per ml of serum responded well to treatment, whereas those with higher viral loads did not. Extensive characterization revealed decreased intrahepatic viral DNA and RNA levels. Liver inflammation was reduced, and woodchuck hepatitis e and surface antigens were cleared from the serum. Significantly, disappearance of intrahepatic core antigen was attributed to a T-cell response against the virus, which was induced by IFN-γ. Importantly, the treatment was shown to be well-tolerated. The same group subsequently evaluated efficacy of IL-12 expressed from a Semliki Forest Virus vector [99]. While the aim was to assess the anti-tumor effects of the constructs in WHV-induced HCC, the authors also showed strong antiviral responses resulting from intrahepatic IL-12 expression. As before, the anti-tumor and antiviral effects were attributed to the induction of T-cell responses. The potential of using cytokines to treat HBV infection was further highlighted by a recent study that evaluated efficacy of dual expression of IFN-α and IL-15 to counter HBV replication in transgenic mice [100]. Co-administration of an AAV encoding the *IFN-α* gene and an AAV carrying the *IL-15* sequence resulted in near complete clearance of intrahepatic HBc and viral DNA replication intermediates. More importantly, IFN-α and IL-15 expression were shown to induce an antibody response and a functional antiviral CD8^+^ T-cell response. The authors further assessed their combination strategy using recombinant IFN-α and IL-15 on samples from patients with chronic HBV infection. After stimulation of peripheral blood mononuclear cells from these patients with an HBc peptide in the presence of IFN-α and IL-15, effector function was restored to HBV specific CD8^+^ T-cells.

Reconstituting the cytotoxic T-cell response against HBV holds great promise as a strategy for the immune-mediated clearance of the virus in chronic carriers. While engineered T-cells with chimeric T-cell receptors against HBV antigens have been developed, investigations are yet to progress beyond preclinical development of the technology [101,102,103,104,105]. DNA vaccines have the potential to induce strong antibody and T-cell-mediated immune responses and thereby clear chronic HBV infections (reviewed by [106]). While studies exploring this strategy are few, inducing strong antiviral immunity with therapeutic vaccines in conjunction with cytokine-mediated stimulation of an immune response undoubtedly has potential for management of chronic hepatitis B.

## 4. Discussion/Perspective

Realization of a functional or complete cure for chronic HBV infections requires innovative therapeutic approaches aimed at disabling and eliminating the persistent episomal cccDNA. Drugs that act directly on the viral genome, such as designer nucleases and epigenetic modifiers, have the potential to disable viral replication permanently. Restoration of the anti-HBV immune response may also facilitate clearance of infected hepatocytes and thus diminish or eradicate the cccDNA pool. A combination of gene-based immune and cccDNA-targeting gene therapy may provide the means to achieve this goal. 

Other challenges, which are broadly associated with implementing gene-based therapies [107], also need to be met for the approach to be successful against HBV. Efficient liver-specific delivery using viral or non-viral vectors still remains a challenge [108,109]. This will be particularly important if multiple doses of gene therapy are required. Improving DNA-binding specificity, particularly for designer nucleases, and defining off target effects are vital to limit unintended side effects [110]. The lack of suitable chronic HBV infection models also complicates the development of gene therapies for the treatment of the disease. The CRISPR/Cas9 system has been shown to target cccDNA efficiently in primary duck hepatocytes infected with DHBV [65]. Although DHBV infection of ducks provides a useful model of chronic HBV infection, it does not recapitulate all aspects of the condition in humans. Despite the challenges facing clinical translation of gene-based curative therapy for chronic HBV infection, the field is gaining momentum and significant progress seems imminent.

## Figures and Tables

**Table 1:** Overview of designer nucleases for HBV gene therapy. RVDs: repeat-variable di-residues; tracrRNA: *trans*-activating crRNA; HDI: hydrodynamic injection; RGN: RNA-guided nucleases

		ZFN	TALEN	CRISPR/Cas
DNA binding domain		Individual ZF proteins recognise nucleotide triplets Typically arranged in arrays of three to four ZFs Heterodimers Targets 18–24 bp	Individual TALE monomer RVDs recognise a single nucleotide Modular assembly of TALE repeats Heterodimers Targets ~40 bp	Single complementary guide RNA Requires PAM and tracrRNA Targets ~20 bp
Nuclease domain		*Fok*I endonuclease fusion protein	*Fok*I endonuclease fusion protein	PAM-dependent Cas protein
Advantages		Naturally occurring mammalian proteins	Easily assembled, highly specific	Very easily synthesized and assembled
Disadvantages		Require arduous context-dependent assembly	Large size limits packaging of both heterodimers into a single delivery vector	Higher potential for off-target cleavage, large Cas proteins limit packaging into delivery vectors
HBV model systems		Huh7 [38] HepAD38 [39]	Huh7 [45,46,47] HepG2.2.15 [45,47] Mouse HDI model [45,46]	Huh7 [50,55,59,63,64,78] HepG2.2.15 [50,51,52,54,56] HepAD38 [49,52,55,64] HepaRG [52] HepG2 [53,54,56,57,59]; HepG2 NTCP * [49,51,54,58,63,64]; HepG2.H1.3 [51]; HepG2.A64 [60,62] Mouse HDI model [50,53,54,56,60,61,78]; Transgenic mice [56,59,60]
cccDNA	Cleavage (%)	No	Yes (12–35%) [45,46]	Yes (10–91%) ** [49,51,54,58,63]
	Reduction (%)	No	Yes (60%) [46] ***	Yes (35–80%) **[50,54,55,56,59,63] [52,64] ***
Alternative effector domain		DNMT3a–catalytic methylation [79] KRAB-transcriptional repressor [80]	KRAB-transcriptional repressor [81]	Cas9 nickase-RGN heterodimer (targets ~40 bp) [51,57]

* Varying methods of introducing the NTCP receptor into HepG2 cells. ** Results from single and/or multiple gRNAs. *** Incorporates co-administration of NAs.
